# Molecular Analysis of a Congenital Myasthenic Syndrome Due to a Pathogenic Variant Affecting the C-Terminus of ColQ

**DOI:** 10.3390/ijms242216217

**Published:** 2023-11-11

**Authors:** Susie Barbeau, Fannie Semprez, Alexandre Dobbertin, Laurine Merriadec, Florine Roussange, Bruno Eymard, Damien Sternberg, Emmanuel Fournier, Hanice Karasoy, Cécile Martinat, Claire Legay

**Affiliations:** 1 CNRS, Saint Pères Paris Institute for the Neurosciences (SPPIN), Université Paris Cité, 75270 Paris, France; 2 INSERM/UEPS UMR 861, Université Paris Saclay, I-STEM, 91100 Corbeil-Essonnes, France; 3 Inserm U 1127, CNRS UMR 7225, Sorbonne Université Paris 06 UMR S 1127, Institut du Cerveau et de la Moelle Épinière, ICM, Sorbonne Université, 75013 Paris, France; 4 AP-HP, Hôpital Pitié-Salpêtrière, 75013 Paris, France; 5 Department of Physiology, Faculté de Médecine Pitié-Salpêtrière, Sorbonne Université, 75006 Paris, France; 6 Department of Neurology, Faculty of Medicine, Ege University, Izmir 35100, Turkey

**Keywords:** ColQ, LRP4, MuSK, AChR, congenital myasthenic syndromes

## Abstract

Congenital Myasthenic Syndromes (CMSs) are rare inherited diseases of the neuromuscular junction characterized by muscle weakness. CMSs with acetylcholinesterase deficiency are due to pathogenic variants in COLQ, a collagen that anchors the enzyme at the synapse. The two COLQ N-terminal domains have been characterized as being biochemical and functional. They are responsible for the structure of the protein in the triple helix and the association of COLQ with acetylcholinesterase. To deepen the analysis of the distal C-terminal peptide properties and understand the CMSs associated to pathogenic variants in this domain, we have analyzed the case of a 32 year old male patient bearing a homozygote splice site variant c.1281 C > T that changes the sequence of the last 28 aa in COLQ. Using COS cell and mouse muscle cell expression, we show that the COLQ variant does not impair the formation of the collagen triple helix in these cells, nor its association with acetylcholinesterase, and that the hetero-oligomers are secreted. However, the interaction of COLQ variant with LRP4, a signaling hub at the neuromuscular junction, is decreased by 44% as demonstrated by in vitro biochemical methods. In addition, an increase in all acetylcholine receptor subunit mRNA levels is observed in muscle cells derived from the patient iPSC. All these approaches point to pathophysiological mechanisms essentially characterized by a decrease in signaling and the presence of immature acetylcholine receptors.

## 1. Introduction

ColQ is a collagen that is expressed and concentrated at cholinergic synapses [1]. In the motor system, it is essentially produced and secreted by muscle cells and preferentially accumulated in the extracellular matrix of the neuromuscular junction (NMJ). A proline-rich domain (PRAD) at the N-terminus of ColQ allows its association with acetylcholinesterase (AChE), an enzyme that hydrolyzes acetylcholine and thus controls the duration of the synaptic transmission. Intracellularly, ColQ and AChE are associated in the endoplasmic reticulum, and the hetero-oligomers are processed in the Golgi apparatus before secretion [2]. AChE exists in multiple molecular forms [3]. At the neuromuscular junction, the main AChE molecular form is called A12, where A stands for asymmetric, and 12 stands for the three tetramers of AChE interacting with the three strands of ColQ. Besides the PRAD, ColQ contains two other domains, a collagen domain with the glycine-X-Y repeated motif characteristic of collagens and a C-terminus domain (CTD). This last domain can be subdivided into two parts: the subdomain after the collagen domain that is necessary for the formation of the triple helix (proximal CTD, P-CTD) and the terminal subdomain (distal CTD, D-CTD) which interacts directly with LRP4 (low-density lipoprotein-receptor-related protein 4) that is complexed with MuSK (Muscle Specific Kinase) forming a synaptic hub at the NMJ [4,5,6,7,8]. Interaction of ColQ with this complex is necessary for the synaptic localization of ColQ, since LRP4 and MuSK are strictly expressed at the post-synaptic membrane, but two heparin binding sites located in ColQ collagen domain are also indispensable for anchorage of the enzyme in the synaptic basal lamina [9].

Congenital Myasthenic Syndromes (CMS) correspond to a group of rare genetic diseases caused by defects in genes expressed at the NMJ. The main, common symptoms are muscle weakness induced by exercise, a decremental electromyographic (EMG) response of the compound muscle action potential (CMAP) after low frequency repetitive nerve stimulation and an early onset of the disease [10]. CMS are classified into pre, post-synaptic or synaptic depending on the specific location of the mutated protein. The CMSs associated to *Colq* variants belong to this last group. In terms of prevalence, these CMSs are among the most frequent CMSs, just behind pathogenic variants in the epsilon subunit of the acetylcholine receptor (AChR). Because no *ACHE* pathogenic variant has been identified and in most muscle biopsies AChE activity is undectable or very low, this CMS is called CMS with AChE deficiency. The groups of Guicheney and Engel showed for the first time a causative link between a CMS with AChE deficiency and a variant in *COLQ* [11,12]. Since then, over 50 pathogenic variants (missense, deletion or nonsense) have been identified in *COLQ*, and these are spread all along the gene, affecting the three major domains described above. The pathogenic variants can either impair the association with AChE (variants in the PRAD) or the formation of the triple helix (variants in the collagen domain or P-CTD). In addition to the defects affecting the previously mentioned domains, some pathogenic variants are present in the sequence coding for the D-CTD and also lead to a more or less severe deficiency in AChE. The missense variant (Y431S) originally identified by the group of Guicheney in 1998 [11] was localized in this domain. Some data suggest that ColQ/MuSK/LRP4 interaction impacts the activity of signalling pathways leading to specific mRNA regulation, the number and the size of AChR clusters, as well as membrane levels of MuSK [13,14]. However, these results were obtained from knock-out mice in the context of a complete deletion of the *Colq* gene. 

The aim of this study was to identify molecular defects induced by variants in ColQ D-CTD that would explain CMSs. For this purpose, we selected a patient with a homozygote frameshift variant that changes part of the sequence of the D-CTD and analyzed its molecular consequences. By combining molecular, biochemical and hiPSC approaches, we provide evidence that the abnormal sequence in D-CTD does not change the structural properties of ColQ but impairs the interaction of ColQ with LRP4 and induces modifications in AChR mRNA levels and the number of AChR clusters. It suggests that the CMS associated with this variant relies at least partially on the reduced interaction between LRP4 and ColQ. It points to the D-CTD as the peptide responsible for LRP4 binding.

## 2. Results

### 2.1. Clinical Case Description

**Figure 1:**
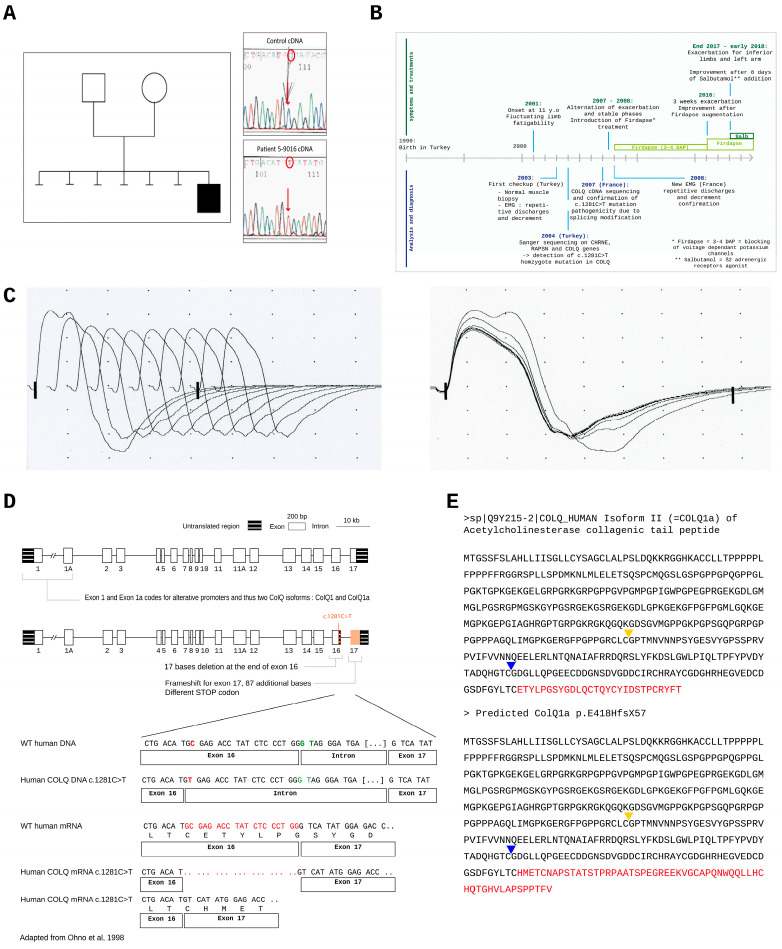
(**A**): Left, pedigree of the family and right, the pathogenic variant identified in the patient cDNA by Sanger sequencing; (**B**): clinical history of the patient; (**C**): EMG showing a decremental response of the compound muscle-fiber action potential (CMAP) after repeated stimulation (left) and the superposed traces (right); (**D**): Intron/exon organization of *COLQ* gene and localization of *COLQ* variant [12]; (**E**): primary sequences of WT and mutated ColQ protein. The yellow arrow head indicates the CTD, the blue arrow head marks the beginning of the distal CTD (D-CTD), the different sequences between normal and ColQ1a p.E428HfsX58 sequences are shown in red.

To understand the role of ColQ D-CTD in molecular interactions, we selected a patient with a homozygote pathogenic variant in the 3′ coding sequence of the gene. This male patient was born in 1990 in Turkey from second degree cousin asymptomatic parents (Figure 1A left panel). He was first examined in 2001 in Turkey where the variant in *COLQ* was first identified by Sanger sequencing (Figure 1A right panel). This variant was then confirmed in France in 2007 where the patient has been since followed (Pitié-Salpetrière Hospital, Paris, France). The patient was born at 32 weeks of gestation. Because of rhesus incompatibility, he received exchange transfusions in the neonatal period. His motor and mental development was normal. Six older siblings died in the neonatal period, probably due to rhesus incompatibility. The clinical history of the patient is represented in Figure 1B. The main characteristics of the patient’s myasthenic syndrome are as follows: onset at 11 years old, fatigable weakness predominant in the limb girdles and the flexors of the neck, and an absence of oculobulbar or respiratory symptoms. Pyridostigmine was ineffective at mitigating the symptoms, whereas 3,4-DAP (45 mg daily), introduced at the age of 18 years, provided a 60% quantified improvement for the patient with walking time increased from 5 to 30 min and a significant reduction in fluctuations. After a long period of stability, at 26 years old, the patient experienced increased weakness of the proximal limbs for 3 weeks, and his dose of 3, 4-DAP was elevated from 45 to 75 mg daily. A year later, a second outbreak of the disease occured, affecting especially the left arm (biceps, wrist and fingers flexors) and lower limbs, with walking time reduced from 20 to 5 min. Treatment with Salbutamol, 6 mg/day, resulted in complete recovery of motor function of the limbs in a few weeks.

Two EMG studies were performed, one at the age of 14 years in Turkey and one at the age of 18 years in France. All showed decremental responses of the compound muscle action potential (CMAP) after repetitive nerve stimulations (3 Hz) (Figure 1C). The amplitude of the decrement was 26% between the 1st and 5th stimulation. Since the amplitude of a decrement is considered as abnormal when higher or equal to 10%, the EMG of the patient is clearly pathological. Deltoid muscle biopsy performed in Turkey revealed normal histological and histochemical features.

Sequence analysis revealed that the patient bears a silent mutation c.1281 C > T that creates a new splice site located 19 nucleotides before the end of exon 16 (Figure 1D) that changes the frameshift of translation. The STOP codon of the normal nucleotidic sequence is not recognized and instead a new STOP codon located 87 nucleotides downstream is used. The translation of this nucleotidic sequence generates a peptide longer by 29 aa than the normal peptidic sequence. The CTD of human ColQ corresponds to aa 294 to 455 and can be subdivided into two subdomains, the CTD domain (for C-terminal trimerization domain) now referred as proximal CTD (P-CTD) from aa 294 to 374 and the distal CTD (D-CTD) from aa 375 to 455. The beginning of the proximal domain is indicated with a yellow arrow and the distal domain with a blue arrow in Figure 1E. In the same figure, the peptidic sequence in red corresponds to the normal sequence and the new sequence generated by the variant. The variant skips part of the WT D-CTD from aa 427, generating a new peptidic sequence (see sequence in red in Figure 1E). About half of the WT distal peptidic sequence is conserved in the mutated sequence. Following the nomenclature of the Human Genome Variation Society (HVGS) for the ColQ protein resulting from this splice site mutation, we refer to the mutated protein as p.E428HfsX58, where p stands for protein, E428H for the first amino acid modified by the mutation in 428 (E) and replaced by an histidine (H), fs for frameshift, and X58 for the first stop codon selected 58 aa downstream the first aa modified (H). For this study, we used the human sequence coding for ColQ1a, the ColQ isoform specifically present at the synapse [15]. Thus, the name of the complete mutated protein is ColQ1a p.E428HfsX58. The genomic coordinates (gDNA level) are the following: Chr3 (GRCh38): g.15453846G > A (cDNA level: NM_005677.4 (COLQ): c.1281 C>T).

### 2.2. The Pathogenic Variant Neither Impairs Transcription nor Translation

**Figure 2:**
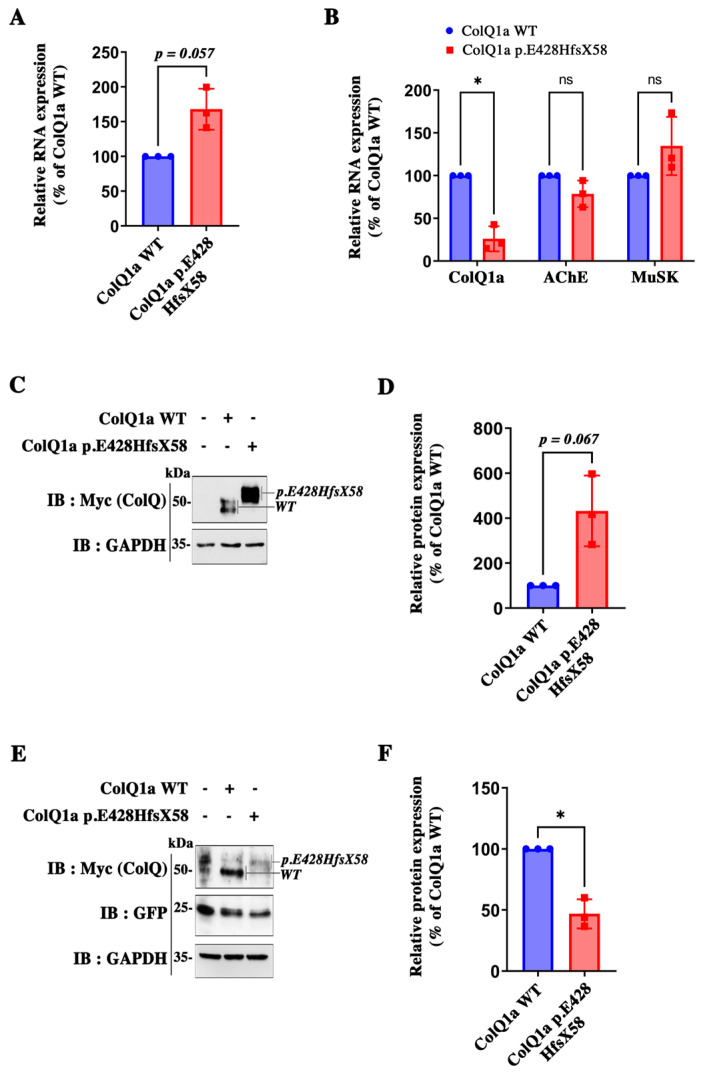
Quantifications of mRNA and protein levels. (**A**): COS cells were transfected with cDNA coding for human ColQ1a WT or ColQ1a E428HfsX58; relative expression of ColQ1a WT and ColQ1a p.E428HfsX58 coding mRNAs were quantified by qPCR. Results are expressed as the mean SD percentage of the ColQ1a WT condition set as 100%. *n* = 3. (**B**): MLCL ColQ-deficient muscle cells infected with *COLQ1a* WT or *COLQ1a* E428HfsX58 coding cDNAs. *COLQ1a*, *Ache* and *Musk* mRNAs were quantified for each condition and results are expressed as above. (**C**): Western Immunoblot of ColQ1a protein expression in COS transfected with *COLQ1a* WT or *COLQ1a* E428HfsX58 cDNAS. (**D**): quantification of data in (**C**). (**E**) Western Immunoblot of ColQ1a protein expression in MLCL ColQ-deficient muscle cells infected with *COLQ1a* WT or *COLQ1a* E428HfsX58 AAVs. and (**F**): quantification of data in (**C**). Results are expressed as the mean ± SD percentage of the ColQ1a WT condition set as 100%. *n* = 3; * *p* < 0.05 using one-sample *t*-test; ns: not significant.

The first question was to determine if ColQ1a p.E428HfsX58 is synthetized. To this end, we investigated the levels of transcripts and proteins. Thus, we performed qPCR to quantify the levels of WT *COLQ* mRNAs compared to those of the *COLQ* variant and western blots to measure the corresponding protein levels. As shown in Figure 2, both the WT and mutated cDNAs were transcribed and translated in COS cells (Figure 2A) and muscle cells (Figure 2B), although at different levels depending on the cell system used for expression. When the expression of *COLQ1a* mutant RNA levels was normalized to the wt, we observed a 63% increase in RNA levels in COS cells (Figure 2A) and a 75% decrease in muscle cells (Figure 2B). The same direction of changes were observed for protein levels between the wt and the mutated proteins with a 4 fold increase in COS cells (Figure 2C,D) and a 54% decrease in muscle cells compared to WT proteins (Figure 2E,F). Since, on one hand, the promoter and the 3′ untranslated sequence are identical for the two constructs and, on the other hand, there is no intronic sequence in any of the two cDNAs, it suggests that a cell-specific transcriptional regulation occurs and involves the mutated sequence. To investigate this possibility, we looked for specific motifs in the DNA sequence; but, when the nucleotidic mutated sequence was blasted (NCBI https://blast.ncbi.nlm.nih.gov/Blast.cgi, accessed on 1 September 2023)**,** no specific regulatory motifs appeared. However, it remains possible that the conformation of the mutated RNA nucleotidic sequence is the target of cell specific stabilizating or destabilizating factors. As expected, the mutated ColQ has a higher molecular weight compared to the WT ColQ in agreement with the sequences (see images of western blots in Figure 2C,E), indicating that the mutated peptide is not cleaved.

**Figure 3:**
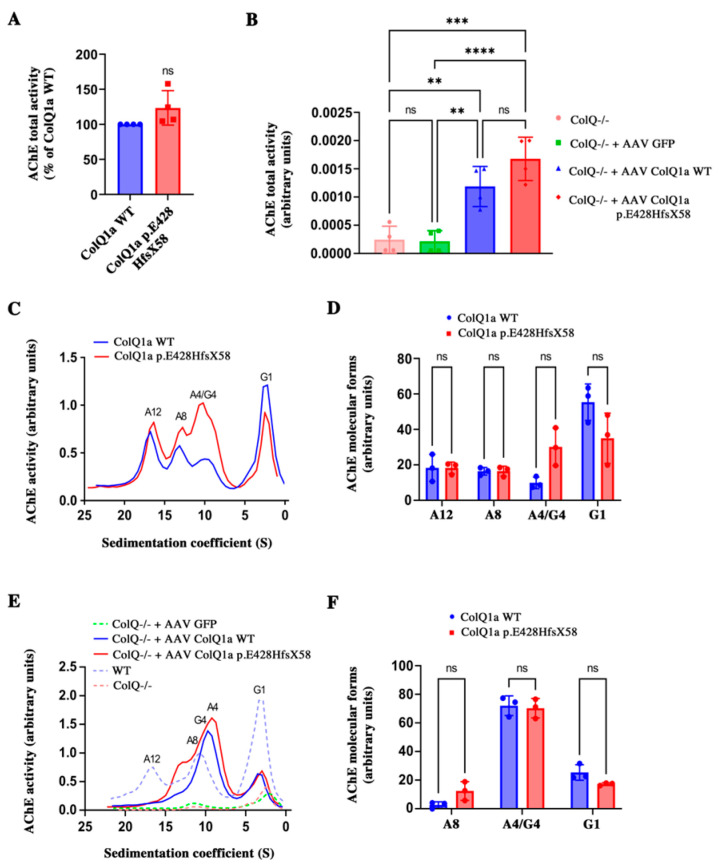
(**A**): Total AChE activity was measured in extracts from COS cells co-transfected with either *Ache* and *COLQ1a* WT or *Ache* and *COLQ1a* p.E428HfsX58 cDNAs. Results are expressed as the mean ± SD percentage of the ColQ1a WT condition set as 100%, using one sample *t*-test; *n* = 4; ns: not significant. (**B**): Total AChE activity measured as above in WT MLCL cells or ColQ-deficient MLCL cells or GFP or *COLQ1a* WT or *COLQ1a* p.E428HfsX58 infected ColQ-deficient MLCL cells. Results are expressed as the mean ± SD using one-way ANOVA followed by Tukey’s multiple comparison post hoc test. (**C**): AChE molecular forms produced by COS cells transfected with *COLQ1a* WT or *COLQ1a* E428HfsX58 cDNAs. (**D**): Quantification of data in (**C**) where for each molecular form results are expressed as the mean ± SD percentage of the ColQ1a WT condition set as 100%. *n* = 3; using one-sample *t*-test. E: Molecular forms produced by WT MLCL cells or ColQ-deficient MLCL cells or GFP or *COLQ1a* WT or *COLQ1a* p.E428HfsX58 infected ColQ-deficient MLCL cells. (**F**): Quantification of data in (**E**) as above. *n* = 2, using one sample *t*-test. ** *p* < 0.01, *** *p* < 0.001, **** *p* < 0.0001; ns: not significant.

To understand the molecular consequences of the pathogenic variant in D-CTD, we performed a detailed analysis of the biochemical properties of the mutated ColQ. We showed above that the ColQ variant was synthetized, but it remained to show that when this protein is associated with AChE, the enzyme is still active. First, we found that the total AChE activity of the WT and mutant hetero-oligomers are similar in COS cells co-transfected with *ACHE* cDNA and either the WT or variant cDNAs (Figure 3A). We then compared WT MLCL muscle cells, ColQ deficient MLCL muscle cells or ColQ deficient MLCL muscle cells infected with either GFP, WT *COLQ1a* or mutated *COLQ1a* AAVs in vitro (Figure 3B). Since muscle cells produce endogenous AChE, we did not co-infect these cells with *Ache* AAV. As previously shown [5], a low AChE activity was detected in ColQ-deficient MLCL muscle cells, and this activity was not different when ColQ-deficient muscle cells were infected with AAV GFP. As expected, enzymatic activity was increased by four-fold when muscle cells were infected with WT *COLQ1a* cDNA and six-fold when cells were infected with p.E428HfsX58 ColQ1a. As in COS cells, no difference in enzymatic activities was observed between ColQ deficient cells infected with the WT construct or the mutated construct. 

Then, we asked if the abnormal peptide could impair the formation of the collagen triple helix and the interaction with AChE tetramers. To answer these questions, we co-transfected COS cells with *Ache* and either WT *COLQ1a* or mutated *COLQ1a* cDNAs and analyzed the profiles of the molecular forms produced by the cells using sucrose gradients. We also infected MLCL muscle cells deficient for ColQ with either GFP, WT *COLQ1a* or mutated *COLQ1a* AAV. As shown in Figure 3C,E, the WT ColQ and mutated ColQ were both able to interact with AChE and generate asymmetric forms (A4, A8 and A12) of AChE in both cell types, although few A^12^ forms were detected in muscle cells whatever the AAV infected. This last observation can be explained by the ratio between endogenous AChE and exogenous ColQ. Indeed, it has been previously shown that the different asymmetric forms are dependent on the ratio between ColQ and AChE [15]. For a given level of ColQ, the more AChE is expressed, the more A12 forms are organized. Quantifications of each of the molecular forms in COS cells and in muscle cells showed no significative difference (Figure 3D,F). Therefore, the abnormal peptide does not introduce any steric hindrance that could perturb the formation of the triple helix. Moreover, AChE and ColQ1a p.E428HfsX58 can associate, producing a panoply of active hetero-oligomers in COS cells and muscle cells. 

### 2.3. The Variant Protein: The Interaction between the ColQ Variant and LRP4 Is Reduced Is Secreted

**Figure 4:**
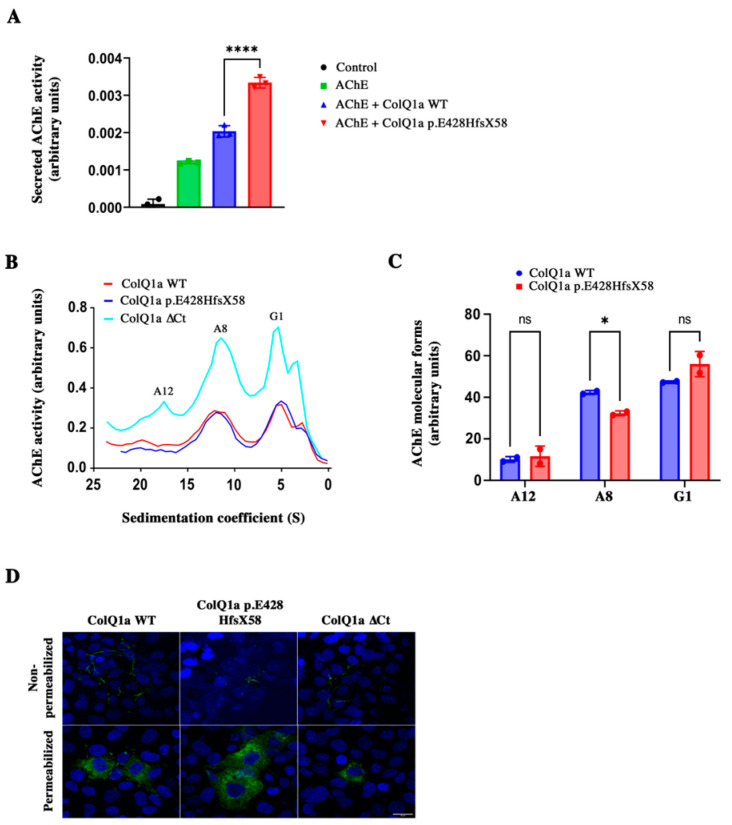
(**A**): secreted total AChE activity in non-transfected COS cells (control) or COS cells transfected with *Ache* alone or *Ache* and *COLQ1a* WT or *Ache* and *COLQ1a* p.E428HfsX58 cDNAs. Results are expressed as mean ± SD one way ANOVA followed by Tukey’s post hoc test; **** *p*<0.0001; ns: not significant (**B**): AChE molecular forms secreted in COS cell medium after co-transfection of *Ache* and WT *COLQ1a* or *COLQ1a* p.E428HfsX58 or *COLQ1a* ∆Ct constructs. (**C**): Quantification of the individual molecular forms secreted in the medium. Data are means ±. SD, one sample *t*-test (*n* = 2); * *p* < 0.05 using one-sample *t*-test; ns: not significant. (**D**): Localization of ColQ by immunohistochemistry after transfection of myc-ColQ1a WT or mutated myc-ColQ1a cDNAs in COS cells without permeabilization or with permeabilization. The tag myc is shown in green. Scale bar, 20 μm.

Can asymmetric forms including ColQ1a p.E428HfsX58 be secreted? To address this question, we quantified the enzymatic activity and the molecular forms in the medium of COS cells expressing AChE and ColQ (WT and mutated). As shown in Figure 4A, WT and mutated hetero-oligomers were secreted after co-transfection of *Ache* and WT or mutated *COLQ1a* cDNA in COS cells. Indeed, enzymatic activity was recovered in COS cell medium after transfections. Both types of hetero-oligomers were more efficiently secreted than AChE alone, indicating that the association of AChE with ColQ stimulates the secretion of the enzyme. However, the level of secreted activity of the mutant hetero-oligomers was significantly higher than the WT hetero-oligomers (Figure 4A). Mostly A8 and G1 (monomers of AChE) forms of WT and mutated hetero-oligomers were found in the medium (Figure 4B,C), although A8 mutated forms were less efficiently secreted than WT forms. For comparison, we transfected a *COLQ* construct deleted from the corresponding mutated D-CTD with *Ache* cDNA. This hetero-oligomer was also secreted, confirming that the ColQ C-terminus peptide offers no impediment to secretion. To confirm the secretion of mutant hetero-oligomers, we performed immunohistochemistry on COS cells transfected with either WT or mutated *COLQ1a* tagged with myc. The localizations of WT ColQ and mutant ColQ were observed in transfected COS cells. No differences were observed for the localization of these proteins in permeabilized or non-permeabilized cells (Figure 4D). In non-permeabilized cells, the myc labeling was observed around the cells, whereas in permeabilized cells, the labeling was mostly observed in intracellular compartments likely to be the Golgi and ER. No higher labeling for the mutated protein compared to the WT one was observed in any compartment. Therefore, the mutated protein does not seem to accumulate in specific organelles. Alltogether, it suggests that hetero-oligomers of AChE/ColQ1a p.E428HfsX58 are secreted. 

### 2.4. The Interaction between the ColQ Variant and LRP4 Is Reduced

**Figure 5:**
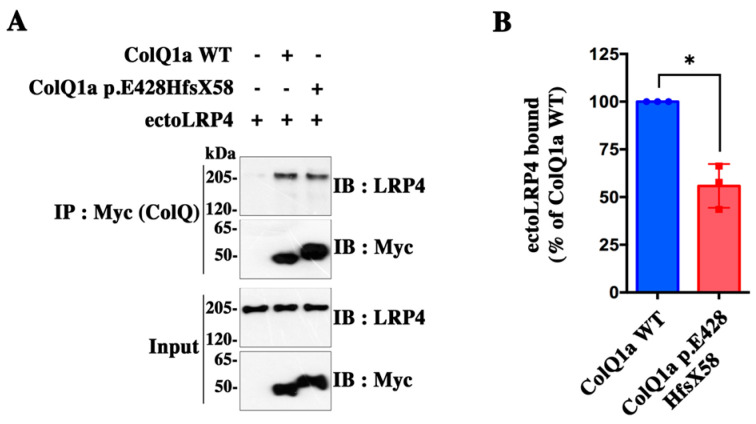
Comparison of LRP4 binding to ColQ1a WT and to ColQ1a p.E428HfsX58 (**A**) Pull-down assay. Magnetic beads coated with similar amounts of Myc-tagged ColQ1an WT and ColQ1a p.E428HfsX58 or ColQ-free beads as a control were incubated with conditioned media of COS cells containing equal amounts of ectoLRP4, as shown in inputs. Pulled down ectoLRP4 was analyzed by Western immunoblot with anti-LRP4 antibodies (**B**) Quantification of ectoLRP4 bound to ColQ1a WT and to ColQ1a p.E428HfsX58 from data in (**A**) reveals that the binding of ectoLRP4 to ColQ1a p.E428HfsX58 was reduced by about 44% compared with ColQ1a WT. Results were normalized to precipitated ColQ1a WT or ColQ1a p.E428HfsX58 and are represented as means ± SD of ColQ1a WT condition set as 100% (*n* = 3; * *p* < 0.05, using one-sample *t*-test).

Recently, we showed that ColQ binds to LRP4 [7]. Thus, we investigated whether the variant in the C-terminus of ColQ might affect the interaction between ColQ and LRP4 using in vitro biochemical methods. For this purpose, magnetic beads were coated with similar amounts of Myc-tagged ColQ1a WT and ColQ1a p.E428HfsX58 and were subsequently incubated with conditioned medium of COS cells containing the same amounts of ectoLRP4 (Figure 5A). After quantification of ectoLRP4 binding and normalization of the results to the amount of ColQ1a WT and ColQ1a p.E428HfsX58 actually linked to the beads, we observed that the binding of ectoLRP4 to ColQ1a p.E428HfsX58 was reduced by about 44% compared with ColQ1a WT (Figure 5B), indicating that the variant in the D-CTD of ColQ significantly alters the interaction between the two proteins. 

### 2.5. iPSC Derived Skeletal Muscle Cells from the Patient Reproduce Some of the Phenotypes Observed in Mice ColQ Deficient Muscle Cells

**Figure 6:**
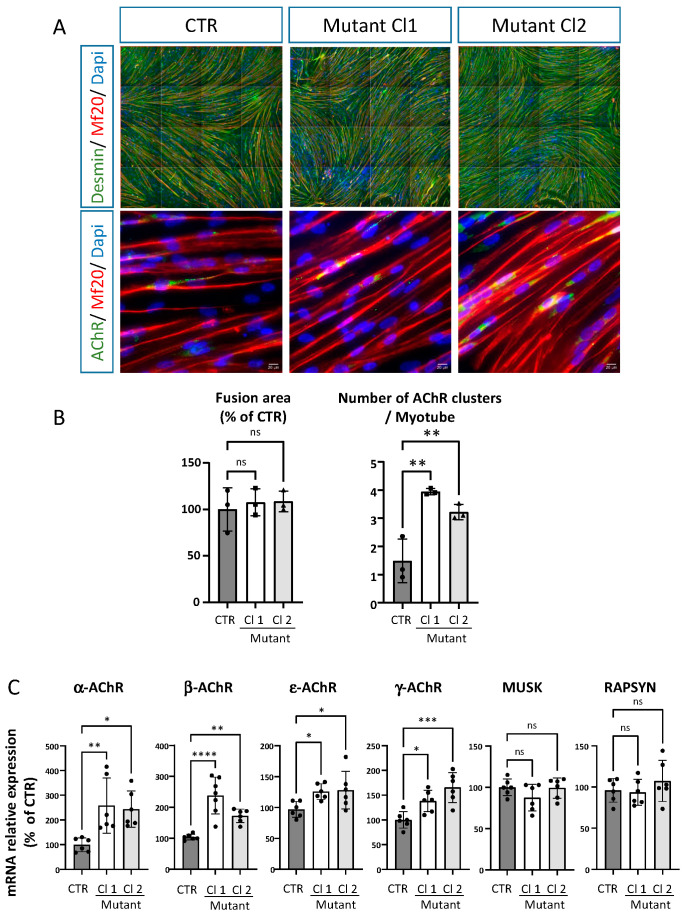
Phenotypic characterization of hiPSC-derived skeletal muscle cells from CTRL and patient. (**A**). Representative images of hiPSC-derived skeletal muscle cells identified by Desmin (green) and Myosin Heavy Chain (Mf20, red) after 5 days of terminal differentiation. Clusters of acetylcholine receptor (green) have been detected after stimulation with Agrin. Scale bar: 20 μm. **(B**). Quantification of the fusion area calculated by Mf20 immunostaining normalized on the number of nuclei. Data represent mean± SD values after normalization on the value obtained with CTRL hiSPC-derived skeletal muscle cells (*n* = 3 independent experiments). The number of AChR clusters per Myotube identified by Mf20 immunostaining was determined on 3 independent experiments in triplicate. Data were analyzed with an ordinary One-Way ANOVA. Tukey’s multiple comparisons tests (*p* > 0.05, ns: not significant, ** *p* < 0,01). (**C**). Quantification of α, β, ε and γ AchR mRNA levels as well as MUSK and RAPSYN mRNA levels by real-time RT-PCR. Level of mRNAs are represented as relative expression (2^−ΔCt^ versus reference gene x100). Data represent the mean ± SD values from three independent experiments in technical triplicate and were analysed with an ordinary one-way analysis of variance (ANOVA), Tukey’s multiple comparisons test compared with CTRL (* *p* < 0.05, ***: *p ≤* 0.001, ****: *p ≤* 0.0001, ns: not significant).

The role of ColQ has been analyzed through the generation of ColQ-deficient mutant mice [16] and a muscle cell line derived from the mutant mice (MLCL: ColQ-deficient cell line) [13,14]. In addition to the lack of asymmetric AChE forms and to reduced AChE activity in the absence of the *Colq* gene in vivo and in vitro, two hallmarks have been observed in vitro in murine muscle cells: 1) AChR clusters are more numerous and smaller; and 2) the levels of mRNAs coding for AChR subunits, MuSK and rapsyn are higher compared to WT muscle cells. We thus wondered if these hallmarks were also present in human muscle cells and if the distal ColQ C-terminus was involved in these molecular phenotypes. To answer these questions, we differentiated skeletal muscle cells from iPSC ones generated from the patient [17]. From the reprogramming, two different clones (Cl 1 and Cl 2) were differentiated into skeletal muscle cells (Figure 6A). Control and iPSC-derived skeletal muscle cells all show the presence of AChR clusters after agrin stimulation. However, the number of AChR clusters was significantly higher in both clones of ColQ mutant hiPS-derived skeletal muscle cells compared to the control cells (Figure 6B). Unlike in mouse ColQ-deficient muscle cells, the area of AChR clusters was similar to that found in control muscle cells. No differences were observed in the fusion area between the control cells and the two clones. The transcripts coding for four AChR subunits (α, β, ε and γ subunits), MuSK and rapsyn were quantified. During development, the γ subunits are replaced by ε subunits in AChR pentamers, a switch that fits with the functional progression of an immature state toward a mature state. In both clones, the levels of AChR subunits coding mRNA were significantly increased, whereas MuSK and rapsyn mRNAs levels were not different from controls (Figure 6C). Thus, AChR transcripts were upregulated in human skeletal muscle cells as in mouse myotubes in the absence of ColQ, which, in both species, also correlate with an increased number of AChR. In this context, it is worth noting that both γ and ε subunit coding transcript levels are increased, suggesting that immature and mature coexist in the human skeletal muscle cells. 

## 3. Discussion

This is the first study which deciphers in-depth molecular mechanisms due to a pathogenic variant causing CMS with AChE deficiency. Using different biochemical methods and various cell systems including iPSC from the patient, we show that the pathogenic variant in the ColQ D-CTD neither affects the association of this mutated ColQ with AChE, nor the formation of the collagen triple helix, nor the secretion of the hetero-oligomer. However, the abnormal peptide generated by the variant significantly reduces the interaction of the mutated ColQ with LRP4. It also leads to an increase of the levels of AChR subunit mRNAs and the number of AChR clusters in iPSC-derived skeletal muscle cells from the patient. 

The main role of ColQ is to bind AChE and to anchor the enzyme in the synaptic cleft through interactions with LRP4/MuSK, perlecan and probably some other proteins of the synaptic basal lamina [5,7,9,18]. These interactions are thought to involve the C-terminal domain (CTD) of ColQ and the Heparin-binding sites present in the triple helix. These domains are both necessary to concentrate the enzyme at the synapse [9]. 

As expected, ColQ1a p.E428HfsX58 is able to associate with AChE since this interaction only requires the PRAD motif in the N-terminus of the protein [19]. There is no evidence that the CTD could be involved in secretion of the hetero-oligomers. The only motif that has been related to AChE secretion is the WAT (tryptophan amphiphilic tetramerization) domain in the C-terminus of AChE, which acts as a retention signal when not associated to ColQ or PRiMA (proline-rich membrane anchor), a structural subunit that anchors AChE in the brain [19,20,21,22]. 

As shown previously in rodents, pathogenic variants in the CTD can lead to the near absence of AChE at the synapse or just to a reduction of AChE activity. These differences are probably related to the location of the variants. Indeed, there is some confusion about the role of the CTD in the literature. The CTD includes two sub-domains with different functions the proximal CTD (P-CTD) as well as the distal end of the CTD (D-CTD). Some splicing variants such as 1082delC mutant truncate part of the P-CTD and the entire D-CTD and therefore lead to mixed defects [12]. But, most of the variants are point mutations in either one of these domains. The P-CTD corresponding to the 80 aa downstream the collagen domain in rats is necessary to form the collagen triple helix [4]. Therefore, variants in this domain are likely to disrupt the proper conformation of ColQ mutants, leading to the degradation of these proteins and thus the absence of AChE at the synapse. Pathogenic variants in this sub-domain are responsible for severe phenotypes. The function of the distal CTD is less clear, and pathogenic variants in this domain generally cause milder CMS (see patients 1, 6, 9, and 12 in [23]). As described earlier, our patient also has a mild phenotype and is now successefully treated with Salbutamol, a b-adrenergic agonist. Interestingly, this drug is effective in most of the CMSs caused by *COLQ* pathogenic variants, no matter the location of the variant. So far, there is no indication of its mechnism of action. It is also beneficial for some patients with CMSs associated to mutations in other genes (MUSK, DOK7, GFPT1, SLC5A7, etc.), suggesting that the targets of the drug are not genetically specific. 

To understand its molecular role, the main approach has been to introduce the sequence of the variants in a WT ColQ coding sequence and express the cDNAs in heterologous cells. In such system, all mutants of the D-CTD including ColQ1a p.E428HfsX58 produce asymmetric forms of AChE. In addition, our study shows that these molecular forms are secreted and should be functional in the synaptic cleft. 

Very recently, Uyen Dao et al. [7] demonstrated that ColQ binds LRP4. They identified the D-CTD as the domain of interaction with LRP4 through two approaches. First, they tested the interaction of a WT peptide containing the last ColQ 27 aa with the ectodomain of LRP4. Second, they used a construct deleted from the entire D-CTD and incubated with the ectodomain of LRP4. They showed that the short 27 aa sequence binds LRP4 and that deleting the entire D-CTD decreases this interaction. Here, the presence of the abnormal peptide in ColQ C-terminus significantly reduces the binding of ColQ1a p.E428HfsX58 to LRP4, confirming that the integrity of the D-CTD is necessary for ColQ interaction with LRP4. Since LRP4 is strictly present at the synapse, the ColQ-LRP4 interaction dictates the synaptic accumulation of AChE. From these results, we can extrapolate that although ColQ1a p.E428HfsX58 is synthetized by muscle cells and asymmetric molecular forms are secreted, the protein might be rather diffusely organized in the synaptic cleft. 

In vivo, the abnormal D-CTD could also impair the interaction with perlecan, but this is very unlikely (1) because perlecan binds to the HBS in the collagen domain and the conformation of the triple helix is normal; and (2) variants in this D-CTD do not affect the interaction with perlecan [18]. It remains possible that in addition to the decrease in binding of the mutated peptide to LRP4, the mutant protein could disrupt the basal lamina by hindering interactions with other proteins and/or collagens as suggested by the group of Maselli [18].

The use of iPSC-derived muscle cells from the patients has allowed us to show that the mutants while expressed in endogenous levels impact the number of clusters and the levels of AChR subunit coding RNAs. This is reminiscent, at least partially, of our prior results [14]. In that study, we looked at the molecular phenotypes of mice-ColQ-deficient muscle cells focusing on the number and the shape of the clusters as well as on the levels of RNAs. The conclusions were that in the absence of the entire ColQ, the number of AChR clusters are increased in agreement with the upregulation of the RNAs coding for the different AChR subunits. In the present study, similar results were obtained, reinforcing the existence of conserved mecanisms in mice and human muscles. However, whereas the preceding results were obtained from cells completely devoided of ColQ, the present study identifies the D-CTD and its interaction with LRP4 as the origin of these mRNA regulations. Here, it should be emphasized that both the γ and the ε subunit coding mRNAs were increased in hiPSC-derived skeletal muscle cells from the patient, suggesting that some AChR clusters are immature, an observation already made previously [14]. Indeed, we have previously shown using immunohistochemistry that the upregulation of the ε and γ subunits correlates with mixed, immature and mature AChR clusters [14]. However, at odds with the results of Sigoillot et al. [14], MuSK and rapsyn mRNA levels were not different from control muscle cells. In mice-ColQ-deficient muscle cells, MuSK transcripts are upregulated, but the levels of membrane-inserted MuSK are lower compared to controls leading to a complex phenotype where AChR clusters are more numerous but smaller. Here, in agreement with normal levels of MuSK transcripts, we did not detect any changes in AChR cluster areas, a result which could be attributed to a species difference. However, it would remain to quantify the membrane MuSK level in human skeletal muscle cells.

In conclusion, our results suggest that the CMS caused by the pathogenic variant of the D-CTD is probably not due to the complete lack of AChE in the synaptic cleft but could be at least partially due to the impaired interaction of ColQ with LRP4 through its D-CTD and putative defects in downstream pathways. Thus, the impaired interaction between the ColQ variant and LRP4 added to the upregulation of transcripts coding for ε and γ subunits would have at least two functional consequences: (1) a more diffuse localization of the enzyme less efficient in the degradation of acetycholine; and (2) the presence of mixed immature and mature AChR clusters. Since AChRs containing γ subunits have a lower conductance, the existence of such AChRs would lower the robustness of neurotransmission [24]. It would therefore be useful to analyze the physiology of the generation of mice bearing this variant. Altogether, our results provide an explanation for the CMS associated to a variant in the D-CTD. Since AChE is still expressed and secreted, it could explain the mild phenotype. The expression of asymmetric forms has also be found for four other variants of ColQ D-CTD [2,11]. It is thus tempting to extend the defective molecular mechanisms revealed by our study to other variants in the same domain. Besides the analysis of the CMS, our results highlight the role of the ColQ terminal domain in signaling. 

## 4. Materials and Methods

### 4.1. Patient and iPSC

The patient is a male referred as 5-9016 (Genethon) who was born in 1990. He signed a written informed consent for his participation in a research program that was approved by a scientific ethical committee (authorization AC-2018-3156). Peripheral blood Mononuclear Cell were purified from a blood sample by Ficoll gradient at the Genethon (Évry, France). HiPSC was generated by the core facility SAFE-iPS at the Institute for Regenerative Medecine and Biotherapy (Montpellier, France). The hiPSC line was named REGUi009-A in agreement with the European nomenclature of the human pluripotent stem cell registry (hPSCreg). For details of the protocol used to generate the iPSC, see Barbeau et al. [17]. The clinical profile of the patient has been briefly described in Wargon et al. [25] and corresponds to patient 2 in this article. Dr. B Eymard (Pitié-Salpétrière Hospital, Paris, France) has followed this patient for the past 15 years after a first diagnosis was made in Turkey by Dr. Karasoy.

### 4.2. Constructs

The rat *Ache* sequence was inserted into the pEFBos plasmid [26]. The patient variant was reproduced in a human cDNA and is referred to as *COLQ1A* E428HfsX58. Human ColQ1a WT-mycTag, Human ColQ1a E428HfsX58-mycTag and Human ColQ1a ΔCt-mycTag were used for co-transfection with *Ache* plasmid. These *COLQ* cDNA sequences were inserted into a pSC2 plasmid. The plasmids were generated by Genscript. The mycTag was inserted after amino acid 24 in the human *COLQ* sequences. For the Human myc-ColQ1a ΔCt plasmid, the nucleotides coding for the last 84 amino acids that correspond to the C-terminal domain of the ColQ1a WT sequence were deleted. For viral infection, the Human ColQ1aWT-mycTag and Human ColQ1a E428HfsX58-mycTag constructs were inserted into AAV viral vectors. A sequence coding for GFP was introduced following an IRES sequence. AAVs were generated by Vector Builder. EctoLRP4 corresponds to the extracellular domain of human LRP4 fused to a 6xHis-tag at the COOH terminus and was generated by Genescript from full length human LRP4 sequence inserted in a pcDNA3 plasmid (gift from Stephan Kröger).

### 4.3. Cell Culture, Plasmid Transfection and Virus Infection

COS cells were maintained in DMEM, 10% FCS, 1% penicillin/strepatavidin and 2 mM L-glutamine at 37 °C in 5% CO_2_. COS cells were either transfected with a cDNA coding for AChE alone or co-transfected with cDNAs coding for AChE and Human ColQ1a WT-mycTag or Human ColQ1a E428HfsX58-mycTag or Human ColQ1a ΔCt-mycTag.

COS cells were transfected at 80% confluence with 5 µg of each plasmid (AChE, Human ColQ1a WT-myc Tag, Human ColQ1a E428HfsX58-mycTag or Human ColQ ΔCt-mycTag) using fuGENE transfection reagent (Promega). Cells were lysed 48 h after transfection for RNA and protein extraction. 

To study the secretion of the AChE-ColQ complex in the culture medium, COS cells were washed twice with PBS 24 h after transfection to eliminate the FCS present in the medium. A new culture medium composed of DMEM, 10% horse serum treated with ISO OMPA (5 × 10^−5^ mol/L; SIGMA) for 1 h at RT to inhibit any exogenous cholinesterase activity), 1% L-glutamine, 1% penicillin-streptomycin and 1 mg/mL heparin (SIGMA) was added to the cells. This culture medium was collected 24 h later for quantification of AChE/ColQ secretion. 

The MLCL WT and ColQ deficient muscle cells lines were used for this study [5]. The myoblasts were cultured in growth medium (GM) made of DMEM, 10% SVF, 20% horse serum, 1% penicillin/strepatavidin, 2 mM L-glutamine, 20U/mL mouse gamma interferon (Sigma) at 33 °C in 8% CO_2_. For differentiation of myoblasts into myotubes, we used a differentiation medium (DM) made of DMEM, 5% horse serum, 1% penicillin/strepatavidin and 2 mM L-glutamine. The cells were seeded in plates coated with collagen type I. This medium was added at 90% of cell confluence. The myotube**s** were cultured at 37 °C in 5% CO_2_.

For viral infection, MLCL ColQ −/− cells were infected 1 day after DM addition when myoblasts start fusing. The infection was performed in serum-free DMEM. Cells were infected with a first dose of virus at 20,000 CG/cell, incubated at 37 °C for 5 h. Then A second dose of virus at 20,000 CG/cell was added and incubated for 2 h. MLCLs were rinsed and further cultured in DM medium. Cells were lysed 4 days after infection for analysis. 

### 4.4. RNA Extraction, Real Time RT-qPCR Assay

Total RNA was extracted from COS cells or muscle cells with the RNeasy kit (QIAGEN). DNase treatment and reverse transcription were realized with the RT2 first strand kit on 1 µg RNA (QIAGEN, Hilden, Germany). Quantitative real-time PCR was performed in triplicate using 10 ng cDNA and the kit Maxima SYBR Green ROX qPCR Master Mix (Thermo scientific, Waltham, MA, USA) using a real-time PCR detection system (Bio-Rad CFX384, South Granville, NSW, Australia). The primers that were used are listed in Table S1 (Supplementary Material). The expression of each gene was normalized to the reference gene Psmd13 (ΔCt). This ΔCt was normalized to a ColQ1a WT control condition (ΔΔCt). RNA expression levels were expressed according to the ΔΔCt method.

### 4.5. AChE Activity and Molecular Form Analysis

*Protein extraction.* For the analysis of the molecular forms synthesized by COS cells cotransfected with AChE and ColQ1a WT-mycTag, ColQ1a E418HfsX-mycTag, or ColQ1a ΔCT-mycTag constructs, three independent experiments were performed. Experiments with MLCL −/− cells infected with ColQ1a WT-mycTag or ColQ1a E418HfsX-mycTag viral constructs were also repeated 3 times. Cells extracts were performed by lysing the cells in Tris base 25 mM, NaCl 0.8 M, EDTA 10 mM, CHAPS 10 g/L, 2 mM Benzamidin and 40 µg/mL Leupeptin. A total of 500 µL of cold extraction buffer was added per 10 cm dish.

*Analysis of the molecular forms in cell extracts and medium*. Cells extracts were centrifuged at 16,000× *g* for 20 min at 4 °C, and the supernatant was recovered for analysis of the molecular forms. For secreted forms, the culture medium was collected and 2 mM Benzamidin and 40 µg/mL of Leupeptin were added.

*Determination of AChE Activity.* AchE activity was determined by the colorimetric method of Ellman et al. [27] using acetylthiocholine as the substrate. Samples (3–10 μL) were added to 200 µL of Ellman assay medium, and the reaction was monitored at 405 nm with a Byonoy Absorbance 96 (byoabs00835). Optical density was recorded at 1 min intervals over a period of 10 min. 

*Molecular form analysis in sucrose gradient.* For sedimentation analyses, 100–800 μL (1DO) samples were mixed with *Escherichia coli* β-galactosidase (16 S) and alkaline phosphatase (6.1 S) as internal sedimentation markers, loaded onto 5–20% sucrose gradients (50 mM phosphate buffer, pH 7.5, 0.8 M NaCl, 10 mM EDTA in the presence of 0.2% Brij-97), and ultra-centrifuged in a Beckman SW41 rotor at 36,000 rpm for 19 h at 4 °C. Approximately 48 fractions of 200 μL were collected and assayed for AChE, β-galactosidase, and alkaline phosphatase activities.

*Determination of β-galactosidase and alkaline phosphatase activities*. The activities of the sedimentation markers, β-galactosidase and alkaline phosphatase were determined by the colorimetric reaction method using ONPG for β-galactosidase and PNPP for alkaline phosphatase as substrates. A total of 50 µL of the first 24 fractions harvested from the sucrose gradients was added to 200 µL phosphate-ONPG buffer and 50 µL of the last 24 harvested fractions was added to 200 µL phospate-PNPP buffer. The optical density was measured at 405 nm with a Byonoy Absorbance 96 instrument (byoabs00835). AChE activity was quantified on 50 µL of each fraction by the colorimetric method previously described. The sedimentation values of the enzymes were calculated according to the positions of the markers, alkaline phosphatase and β-galactosidase. 

### 4.6. Recombinant Protein Production and Purification

For the production of ectoLRP4, the cell culture medium (DMEM 10% FCS) of transfected COS cells was replaced 24 h after transfection with Opti-MEM reduced serum medium (Fisher Scientific) supplemented with 0.01% of FCS. Cells were grown at 32 °C to increase the secretion of ectoLRP4. The next day, media were collected, concentrated 30-fold using Amicon Ultra-30 centrifugation filters (Millipore) and stored at 4 °C in the presence of protease inhibitors and 0.05% sodium azide.

Pull-down assay: Myc-tagged ColQ1a WT or ColQ1a p.E428HfsX58 from transfected COS cell lysates was immobilized for 2 h on protein G magnetic beads (Dynabeads) precoated with anti-Myc antibodies. Control beads correspond to beads precoated with anti-Myc that were incubated with the same protein concentrations of COS cell lysates from non-transfected cells. The beads were then washed and incubated with conditioned medium containing ectoLRP4 at 4 °C overnight. Beads were washed 3 times with lysis buffer and 1 time with 50 mM Tris buffer. Bound proteins were eluted with 1× Laemmli sample buffer at 70 °C and analyzed by western immuniblot. 

### 4.7. Western Blot Analysis

Protein extraction from cultured cells was performed in 250 µL of RIPA buffer (tris base 50 mM, NaCl 150 mM, EDTA 3 mM, NaF 20 mM, Triton 1%, DOC 0.5%, SDS 0.1%, pH 7.5) for a 10 cm plate, anti-proteases (Roche, Basel, Switzerland,) and anti-phosphatases (Roche, Basel, Switzerland). The lysates were incubated in ice for 2 h under continuous stirring, then centrifuged at 16,000× *g* for 20 min at 4 °C. Protein extracts were assayed with the BCA colorimetric method (Uptima, Pittsburgh, PA, USA). Protein concentrations were determined using a BSA calibration range (Interchim). The optical density was measured at 570 nm with a Byonoy Absorbance 96 instrument (byoabs00835).

For the western blot, protein extracts were denatured in Laemmli buffer (BioRad, South Granville, NSW, Australia) and β-mercaptoethanol at 95 °C for 10 min; then, they were resolved on 8% Bis tris polyacrylamide gel (Thermo Scientific, Waltham, MA, USA) in MOPS SDS running buffer (Thermo scientific, Waltham, MA, USA) and transferred to nitrocellulose membranes (Millipore, Burlington, MA, USA) in Tris glycine transfert buffer (BioRad) before incubation with primary antibodies in blocking buffer (Thermo Scientific, Waltham, MA, USA) overnight at 4 °C. All the antibodies are listed in the Table S1 in the Supplementary Material. After several washings, the membrane was incubated with HRP-conjugated secondary antibodies for 1 h at room temperature. The proteins were visualized using chemiluminescence (Cytiva, Marlborough, MA, USA, ECL prime) and quantified using ImageJ’s gel analysis.

### 4.8. Immunodetection and Microscopy

Cells were fixed with 4% PFA for 10 min. After several washings, they were incubated in blocking buffer (Abcam, Cambridge, USA) for 1 h at room temperature. Primary antibodies were diluted in the blocking buffer, and cells were incubated with these antibodies overnight at 4 °C (see Table S1 in Supplementary Material). Four washes in PBS-tween 0.1% were performed. Secondary antibodies were diluted in the blocking buffer and added to the cells for 1 h at room temperature. Nuclei were visualized with DAPI (Invitrogen, Waltham, MA, USA) diluted 1:5000 in PBS. At the end, four washes with PBS-tween 0.1% were performed. The prolong Diamond antifade mountant (Invitrogen, Waltham, MA, USA) was used for mounting the cells. The stainings were observed using the confocal microscope ZEISS LSM 880 airyscan or epifluorescence microscope NIKON TE2000. The analysis of the microscopy images was performed with image J.

### 4.9. Differentiation of iPSC into Myoblasts

hiPSCs skeletal muscle differentiation experiments were performed using the commercially available Stemcell Technologies®, Vancouver, Canada, skeletal muscle differentiation media and adapted from a protocol described previously [28]. Briefly, hiPSC cells were dissociated with Stem Pro Accutase Cell Dissociation (Gibco^®^, Waltham, MA, USA) and differentiated using the MyoCult-differentiation kit (Stemcell technologies^®^, Vancouver, Canada). On day 30, myoblasts were dissociated and cryopreserved. For terminal differentiation, cells were thawed at 30.000 cells per cm^2^ in MyoCult-SF expansion medium for 2 days (Stemcell technologies^®^, Vancouver, Canada). Then, the medium was switched to MyoCult Differentiation medium for 5 days. For AChR clustering analyses, cells were treated for 6 h with agrin at 0.5 μg/mL (R&D systems). After fixation with 4% PFA for 10′ at room temperature, cells were incubated for 1 h at room temperature with the following primary antibodies: DESMIN (R&D System^®^, Minneapolis, MN, USA, 1:100), MF20 (DSHB^®^, Iowa City, Iowa, 1:200) and AChR (DSHB^®^, Iowa City, Iowa, 1:100). Appropriated Alexa fluorescent secondary antibodies (Invitrogen^®^, Waltham, MA, USA, 1:1000) and Hoechst (5 μg/mL) were added for 1 h. Image acquisitions were performed on the spinning disk confocal (Zeiss®, Jena, Germany) with the 63X objective and CellInsight High content CX7 system (ThermoFisher^®^, Waltham, MA, USA) with the 20X objective. Images were analyzed using Fiji Software (version v1.54f, Zurich, Switzerland) and HCS Studio software as previously described [29,30]. For RTqPCR, total RNA was extracted using the RNeasy Micro/Mini kit (Qiagen®, Hilden, Germany) and was reverse transcribed using random hexamers and the Superscript III Reverse Transcriptase kit (Invitrogen^®^, Waltham, MA, USA). Quantitative PCR reactions were carried out in 384-well plates using a QuantStudio 12K Flex Real-Time PCR System (Applied Biosystems^®^, Waltham, MA, USA) with Power SYBR Green 2 × Master Mix (Life Technologies®, Carlsbad, CA, USA), 2.5 μL of cDNA and primers (listed below) in a final volume of 10 μL. The 2^−ΔΔCt^ method was used to determine the relative expression level of each gene.

## Data Availability

The graphs and statistics were generated with the graphPad Prism (Prism 9, version 9.5.1, 26 January 2023, California, USA). All data are means ± SDs.

## Supplementary Materials

The following supporting information can be downloaded at: https://www.mdpi.com/article/10.3390/ijms242216217/s1.
