# Supplementary material for: Molecular Analysis of a Congenital Myasthenic Syndrome Due to a Pathogenic Variant Affecting the C-Terminus of ColQ
Source: Int J Mol Sci. 2023 Nov 11;24(22):16217. doi: 10.3390/ijms242216217 (PMC10671321; doi:10.3390/ijms242216217)
Supplement: Supplementary file 1 [file ijms-24-16217-s001.zip › ijms-2665588-supplementary.pdf]

Resource		
**Primary antibodies**	**Source**	**references**
Anti-alpha tubulin	sigma	T5168
Anti-GAPDH	Abcam	ab8245
Anti-GFP	Roche	11814460001
Anti-MuSK	millipore	ABS549
Anti-Myc	cell signaling	2276S
Human Anti-Nicotinic acetylcholine receptor	abcam	ab65180
Mouse monoclonal anti-LRP4 clone S207-27	sigma	SAB5200690
**secondary antibodies**		
Alpha bungarotoxin conjugate alexa fluor 488	invitrogen	B13422
Alpha bungarotoxin conjugate alexa fluor 555	invitrogen	B35451
Alpha bungarotoxin conjugate alexa fluor 594	invitrogen	B13423
IgG (H+L) Cross-Adsorbed Goat anti-Mouse, Cyanine3	invitrogen	A10521
IgG (H+L) Cross-Adsorbed Goat anti-Rabbit, Cyanine3	invitrogen	A10520
IgG (H+L) Highly Cross-Adsorbed Goat anti-Mouse, Alexa Fluor 594	invitrogen	A11020
IgG (H+L) Highly Cross-Adsorbed Goat anti-Mouse, Alexa Fluor 633	invitrogen	A21052
IgG (H+L) Highly Cross-Adsorbed Goat anti-Rabbit, Alexa Fluor 488	invitrogen	A11008
IgG (H+L) Highly Cross-Adsorbed Goat anti-Rabbit, Alexa Fluor 594	invitrogen	A110212
IgG (H+L) Highly Cross-Adsorbed Goat anti-Guinea Pig, Alexa Fluo 633	invitrogen	A21105
IgG (H+L) Cross-Adsorbed Goat anti-Rat, Alexa Fluor 633	invitrogen	A21094
Rabbit IgG HRP Linked Whole Ab	Sigma	NA934V
Mouse IgG HRP Linked Whole Ab	Sigma	NA931V
**Oligonucleotide primer sequences**		
Human_COLQ_2_SG QuantiTect Primer Assay	Qiagen	QT01155588

RT² qPCR primer Assay for human GAPDH	Qiagen	PPH00150F-200
RT² qPCR primer Assay for mouse AChE	Qiagen	PPM35356A-200
RT² qPCR Primer Assay for Mouse Musk	Qiagen	PPM25832B-200
RT² qPCR Primer Assay for Mouse Psmd13	Qiagen	PPM27628A-200
**Oligonucleotide primer sequences**	**Forward**	**Reverse**
18S	GAGGATGAGGTGGAACGTGT	TCTTCAGTCGCTCCAGGTCT
CHRNA	TGAAATTCGGCTCCTGGACC	GCACAGCACTCGTACTTCCT
CHRNB	GTCCACGAACGGAACTTCAT	CTATTCCAATGCCGTGGTCT
CHRNE	GTCTCCATCAACGTCCTGCT	GTTGAGCACGATGACGCAAT
CHRNG	ATCGTGCTGGAGAACAACGT	ACTGAGATAGAGCAGGCGGA
MUSK	ACCAATGACGTCCTCAAAGC	TCAGAAGGCAGTGTGGTGAG
RAPSYN	CTGCACTGTCTGAGCGAGAG	ACCTGAGGTGGAAGATGTGG

Table : List of antibodies and primers used qPCR
